# Preclinical Evaluation of a New Surgical Hybrid Energy Device Compared to the Conventional Energy Device

**DOI:** 10.1111/ases.70201

**Published:** 2025-11-27

**Authors:** Satoru Matsuda, Kazuhisa Ehara, Hiroyasu Kagawa, Shunsuke Tsukamoto, Kohei Nakata, Mamoru Morimoto, Sayaka Yasui, Jumpei Torikai, Ichiro Uyama

**Affiliations:** ^1^ Department of Surgery Keio University School of Medicine Tokyo Japan; ^2^ Department of Gastrointestinal Surgery, Gastric Surgery Division Saitama Cancer Center Saitama Japan; ^3^ Department of Gastrointestinal Surgery Institute of Science Tokyo Tokyo Japan; ^4^ Department of Colorectal Surgery National Cancer Center Hospital Tokyo Japan; ^5^ Department of Surgery and Oncology, Graduate School of Medical Sciences Kyushu University Fukuoka Japan; ^6^ Division of Surgery Keiyu‐Kai Sapporo Hospital Sapporo Japan; ^7^ Olympus Medical Systems Corp Tokyo Japan; ^8^ Department of Surgery Fujita Health University School of Medicine Toyoake Japan

**Keywords:** endoscopic surgery, energy device, surgical instrument

## Abstract

**Introduction:**

Despite the development of surgical instruments combining ultrasonic scissors and an advanced bipolar device, thermal management has still been considered a persistent issue, similar to that in conventional ultrasonic scissors. To address this issue, the next‐generation hybrid device (NGHD) with a new tip and handle design has been devised while maintaining the high cutting and sealing performance of combined surgical instruments (THUNDERBEAT Type S, TBS). We compared them in an ex vivo and nonclinical in vivo environment and investigated their perceived clinical usefulness.

**Methods:**

Ex vivo testing included measurements of cutting speed, posterior thermal spread from the jaw, and lateral thermal spread. The nonclinical in vivo study involved evaluations such as cutting capability while maintaining the tissue layer, and handle ergonomics.

**Results:**

The NGHD demonstrated a significantly faster cutting speed than did TBS (2.41 [2.31–2.50] vs. 3.04 [2.94–3.13] s, *p* < 0.01) with 5–7 mm arteries, as well as significant improvements in the posterior thermal spread on arteries during activation (5.67 [0–12.84] vs. 84.22 [35.34–133.10] μm, *p* < 0.01) and lateral thermal spread (2.22 [2.06–2.38] vs. 2.79 [2.55–3.03] mm, *p* < 0.05). Surgeon assessments indicated more improvements with the NGHD than with TBS, especially regarding cutting while maintaining the tissue layer and handle ergonomics.

**Conclusion:**

The NGHD promoted improvements in thermal management generally associated with TBS and maintained the benefits of combining devices capable of ultrasonic and advanced bipolar output. The NGHD has the potential to support safe and efficient endoscopic procedures, pending further clinical evaluation.

## 1 Introduction

With the recent advancements in the development of surgical instruments, endoscopic surgery has become widely adopted as one of the standard surgical procedures in various fields. Minimizing surgical injury and magnifying the surgical view help surgeons reduce blood loss and perform precise surgical manipulations, including lymph node dissection. Recent studies have reported on the advantages of endoscopic approaches in gastrointestinal cancer surgery. Moreover, evidence suggests that laparoscopy was not inferior to open surgery in gastric cancer surgery [1, 2]. In recent years, studies have also demonstrated that laparoscopy was not inferior to open surgery in terms of overall survival in esophageal cancer surgery [3, 4]. Considering these advantages, the frequency of endoscopic surgeries has been increasing worldwide. Indeed, the National Clinical Database of Japan showed that in 2021, half of all gastrointestinal cancer surgeries were performed via the endoscopic approach [5]. The percentage was particularly high for rectal and esophageal cancers, at around 75% in the NCD, similar to that observed in Western Societies [6, 7].

To improve the quality of endoscopic surgery, advancements in surgical instrumentation are essential. In particular, dissecting devices have been found to directly affect the amount of blood loss, postoperative complications, and the quality of lymph node dissection. Aside from electrocautery, the development of ultrasonic scissors and advanced bipolar devices has strongly encouraged the widespread use of endoscopic surgery. Ultrasonic scissors allow for precise manipulation during surgery owing to their fast incision speed and the ability to incise to the tip. However, concerns regarding tissue damage caused by cavitation at the tip of the instrument [8], the mist resulting from the cavitation, thermal damage due to the heat of the device, tissue pad durability [9], and hemostatic properties in larger vessels have been reported. Advanced bipolar devices are relatively better than ultrasonic scissors for hemostasis but have a longer cutting time and concerns regarding lateral thermal spread [10].

To overcome these concerns while maintaining its advantages, a combined surgical instrument comprising ultrasonic scissors and an advanced bipolar device has been devised. The addition of the advanced bipolar function to the ultrasonic scissors successfully increased the hemostatic force while maintaining cutting speed and capability for meticulous dissection [11]. However, the lateral thermal spread from the device, the posterior thermal spread from the back of the jaw of the device [12], and the tissue pad durability have continued to be an issue of TBS, similar to that observed with conventional ultrasonic scissors.

To address these remaining issues, the next‐generation hybrid device (NGHD) had been devised. The new tip design of the NGHD is expected to reduce the thermal spread to the surrounding organs, improve the durability, and maintain the basic sealing and cutting capability. Furthermore, the handle of the NGHD was designed for improved ergonomics compared to the conventional device. We then compared the current and next‐generation combined devices in an ex vivo and nonclinical in vivo environment and investigated their perceived clinical usefulness.

## 2 Materials and Methods

### 2.1 Surgical Instruments

**FIGURE 1:**
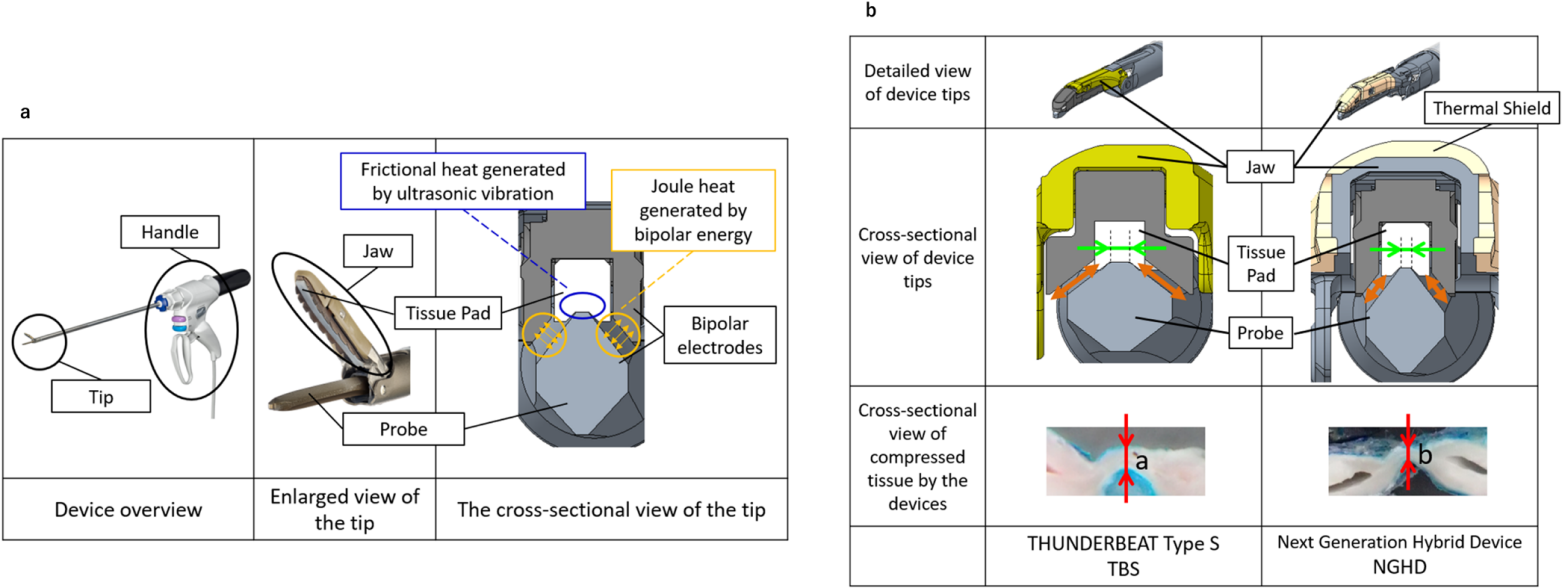
(a) Definition of each part of the devices and the mechanism of the SEAL & CUT Mode. (b) Overview of the tip of the hybrid devices. The NGHD allows for greater tissue compression than would TBS owing to the structure of its tip, which indicates that (b) is thinner than (a) in the cross‐sectional view of the tissues compressed by the devices. Green arrow: width of the top surface of the probe; Orange arrow: area of the bipolar electrode. Abbreviations: NGHD, next‐generation hybrid device; TBS, THUNDERBEAT type S.

The performance of the NGHD (Figure 1a) was compared to that of THUNDERBEAT type S (TBS) (Olympus Medical Systems Corp., Tokyo, Japan), a surgical instrument that combines ultrasonic scissors and an advanced bipolar device. The burst pressure of NGHD was also compared to that of POWERSEAL (PS) (Gyrus ACMI Inc., MN, United States), a representative model of an advanced bipolar device.

The NGHD and TBS featured a SEAL & CUT Mode (S&C) that combines ultrasonic and advanced bipolar output and a Seal Mode capable of advanced bipolar output. S&C enables fast tissue cutting with vessel sealing by combining frictional heat generated by ultrasonic vibration and joule heat generated by bipolar energy (Figure 1a). To achieve the combined output, several interrelated parameters, such as mechanical dimensions, shape, physical interactions, and electrical characteristics, need to be precisely adjusted. Hence, to ensure excellent performance, usability, and durability all at the same time, the parameters of the NGHD were tuned.

One key parameter is grasping force. To achieve strong vessel sealing and cutting performance, both the ultrasonic scissors and advanced bipolar device need to be capable of appropriate tissue compression. By slimming down the design of the probe and jaw, specifically by narrowing the top surface of the probe (green arrow in Figure 1b) and sharpening the slope angle (orange arrow in Figure 1b), the NGHD was able to concentrate the grasping force toward the center of the jaws. This design allows more efficient transmission of pressure to the tissue, resulting in improved compression compared to the TBS (Figure 1b, cross‐sectional view of the tissue compressed by the devices). All related parameters were adjusted to align with the slimmer design and intended performance.

By slimming the probe and jaws to concentrate the grasping pressure at the center and by optimizing various design parameters, more efficient energy transfer to the tissue was achieved. Consequently, the amount of energy required for each activation was reduced, especially the amplitude of the ultrasonic probe (NGHD: up to 67 μm; TBS, 80 μm) and the area of the bipolar electrodes (orange arrows shown in Figure 1b). Overall, these design changes in the NGHD resulted in several advantages over TBS, such as faster cutting while maintaining the tissue layer, comparable vessel sealing, reduced lateral thermal spread, reduced cavitation due to decreased ultrasonic amplitude, and enhanced tissue pad durability.

Also, the jaw of the NGHD is covered with resin that slows down heat transfer (thermal shield in Figure 1b), which reduces thermal damage to the tissues when the device comes in contact with the surrounding tissue during and after output. Other than that, there are no changes from TBS to NGHD, such as the high‐frequency output characteristics and materials of the tip.

The NGHD and TBS have three output settings for S&C that deliver different cutting speeds: level 1, 2, and 3. The cutting speed of S&C of NGHD increases as the level rises, while the cutting speed of the S&C of TBS decreases as the level increases, respectively, due to the product specifications. In this study, the fastest cutting settings, namely S&C level 3 for NGHD and S&C level 1 for TBS (Table 1), were selected for each device.

**TABLE 1:** Output mode specification for the NGHD and TBS.

Output mode	NGHD	TBS
SEAL & CUT	Output Setting: Levels 1–3 The higher the output level setting, the faster the cutting speed.	Output Setting: Level 1–3 The lower the output level setting, the faster the cutting speed.
SEAL	Output Setting: Not adjustable	Output Setting: Not adjustable

Abbreviations: NGHD, next‐generation hybrid device; TBS, THUNDERBEAT type S.

### 2.2 Bench‐Top Test

All organs used in the following tests (cutting speed, burst pressure, thermal spread, tissue pad durability, and cavitation) were obtained from domestic pigs. These organs were harvested and prepared on the previous day and stored in refrigeration. Prior to bench‐top testing, organs were warmed to body temperature using a water bath. Table 2 summarizes the outer diameters of the blood vessels and lymphatic vessels, as well as the organs from which they were extracted.

**TABLE 2:** Outer diameters of the blood and lymphatic vessels, as well as the organs from which they were extracted.

Stated diameter	Definition (*X*: diameter)	Organs
5 mm or smaller	*X* ≤ 5	Porcine splenic and carotid arteries
3–5 mm	Greater than 3 mm but less than or equal to 5 mm (3 < *X* ≤ 5)	Porcine carotid arteries
5–7 mm	Greater than 5 mm but less than or equal to 7 mm (5 < *X* ≤ 7)	Porcine renal arteries
2–4 mm	Greater than 2 mm but less than or equal to 4 mm (2 < *X* ≤ 4)	Porcine thoracic duct

### 2.3 Cutting Speed

Cutting time was measured in 7 mm or smaller arteries using the NGHD at S&C level 3 and TBS at S&C level 1. The duration from the start of the output to complete separation of the blood vessels was then measured (Figure S1). The acquired data consisted of 60 samples from 5 mm or smaller arteries and 30 samples from 5 to 7‐mm arteries.

### 2.4 Burst Pressure

Burst pressure was measured in 5‐mm or smaller arteries (*n* = 120 per device), 5–7‐mm arteries (*n* = 60 per device), and 2–4‐mm lymphatic vessels (*n* = 30 per device) using the NGHD at S&C level 3 and TBS at S&C level 1, as well as in 5–7 mm arteries (*n* = 30) using PS. Burst pressure was measured by injecting normal saline into sealed blood vessels and continuing the measurement until leakage of normal saline was detected from the edge excised by sealing and cutting (Figure S1). The probability of burst pressure exceeding 360 mmHg was calculated using the cumulative density function. In vessel sealing energy devices, it is common to evaluate sealing performance using probability [13].

Furthermore, to confirm the burst pressure value upon the application of physical stimulation, such as wiping blood and tissue fluid around the sealed area with a gauze, the following test was conducted. Accordingly, 3–5‐mm arteries were sealed and cut using the NGHD at S&C level 3 and TBS at S&C level 1. After sealing and cutting the blood vessels, the edge excised by applying S&C was wiped horizontally and vertically using a gauze with 3 or 6 N of force. The burst pressure for the samples whose edge was wiped with gauze was measured using the same method described earlier in this section, after which the probability of the burst pressure exceeding 360 mmHg was calculated. For each condition, 30 samples were obtained.

### 2.5 Thermal Spread

Both posterior thermal spread, which occurs when the back of the jaw comes into contact with the tissue, and lateral thermal spread, which occurs across the tissue when the device was applied, were tested. During these tests, the NGHD at S&C level 3 and TBS at S&C level 1 were used.

Posterior thermal spread into the tissue was tested using the following three patterns (Figure S2):

1. The back of the jaw was placed in contact with the tissue after output completion with full‐length bite for 10 times.
2. A single output with full‐length bite was performed while the back of the jaw was in contact with the tissue.
3. Several outputs with three different grasping conditions: full‐length bite, half bite, and tip bite, were performed by placing the back of the jaw in contact with the livers until thermal damage was confirmed.

The tissue used to output the device was the small intestinal mesentery, whereas the tissues used for contact with the device to confirm posterior thermal spread were the small intestines, livers, carotid arteries, and renal arteries. As for No. 1 and No. 2, the tissue that suffered posterior thermal spread was subjected to histological analysis (for details regarding the methods used, refer to Section 2.6). In these tests, 9 samples each from the small intestine and liver, 7 samples from the carotid arteries, and 16 samples from the renal arteries were collected. As for No. 3, the posterior thermal spread was confirmed visually. The output simulated three tissue grasping conditions: full, half, and tip grasping. The number of outputs until thermal spread occurred was measured, with three samples being collected for each condition.

The lateral thermal spread was measured by applying the device with full‐length bite to the mesometrium uteri (*n* = 40 per device). The white burn area on the organ caused by the application of the device was measured visually using image analysis software (Image J version 1.54d) (Figure S3).

To support the thermal damage data, the temperature of the device tip was also measured by the thermographic camera (*n* = 30 per device). The temperature of the back of the jaw was measured after output completion with a full‐length bite 10 times.

### 2.6 Histological Analysis

For histological analysis, tissues were fixed with 10% neutral‐buffered formalin immediately after device application. The samples were embedded in paraffin, sectioned, and stained with hematoxylin and eosin. The range of thermal spread was then measured by analyzing the extent of cell degeneration on stained samples. Thermal spread measurements were performed on stained samples using objective lenses ranging from 10× to 20× magnification.

To preserve the objectivity of the data, the individual responsible for creating samples (the evaluator) and the members conducting the measurements of samples were separate and assessed in a blinded fashion.

### 2.7 Tissue Pad Durability

The durability of the tissue pad attached to each device was tested by repeating the following tasks: cutting the small intestinal mesentery with full‐length bite and activating the devices (NGHD at S&C level 3 and TBS at S&C level 1) for 1–2 s after completing the cutting procedure without the tissue between the pad and the probe. The task was repeated until an error occurred due to pad damage, after which the cumulative number of tasks was recorded (Figure S4). Three samples were obtained for this test.

### 2.8 Cavitation

The extent of cavitation generated by ultrasonic vibrations from the devices and the maximum distance of cavitation were measured. After placing the tip of the device in normal saline, the NGHD at S&C level 3 and TBS at S&C level 1 were activated, and the cavitation generated thereafter was recorded using a high‐sensitivity camera. The distance of cavitation diffusion in normal saline and the extent of cavitation, determined as the percentage of cavitation in a given area of normal saline, were measured (Figure S5). Eight samples were obtained for each measurement.

### 2.9 Nonclinical In Vivo Evaluation

All procedures performed in vivo, along with the animals used in this test, were reviewed and approved by the Institutional Animal Care and Use Committee at a Japan Pharmaceutical Information Center‐accredited facility in accordance with the Basic Policy on Animal Experimentation Performed at the Institutions under the Jurisdiction of the Ministry of Health, Labour, and Welfare.

Seven surgeons simulated the clinical use of the NGHD and TBS by incising the peritoneal membrane and membrane tissue around the stomach, as well as the mesentery and membrane tissue around the intestines, in four live female pigs (58–61 kg), to assess the perceived clinical usefulness of the NGHD. Subsequently, seven items (posterior thermal spread from the jaw, lateral thermal spread, cutting while maintaining the tissue layer, tip visibility during the procedures, handle ergonomics, mist/cavitation, and sealing and cutting performance) were evaluated qualitatively on a 5‐point scale for each device.

### 2.10 Statistical Analysis

Statistical analysis was performed using the Minitab 21 statistical software package (Minitab Inc.) and the IBM SPSS Statistics 26 software package (SPSS Inc.). Normality was comprehensively assessed based on both quantitative results obtained through the Shapiro–Wilk test and qualitative results based on data distribution. Based on these results, two‐sample *t*‐tests or Mann–Whitney tests were performed to test for significant differences between the two groups. Significant differences between three or more groups were determined using Gomez–Howell pairwise comparisons, Tukey's HSD test, or Dunn's test. In all tests, the level of significance was set at 0.05 on both sides. For all tests, the significance level was set at 0.05 for two‐tailed tests. Additionally, for groups showing statistically significant differences, effect sizes were calculated using Cohen's *d* or Cliff's delta [14].

All the data were verified by academics, Ichiro Uyama, Satoru Matsuda, Kazuhisa Ehara, Hiroyasu Kagawa, Shunsuke Tsukamoto, Kohei Nakata, and Mamoru Morimoto, independently.

## 3 Results

Descriptive statistics for the results of each test were summarized in Table 3.

**TABLE 3:** Summary of bench‐top test outcomes.

Evaluation items	Devices	*N*	Mean [95% CI]	SD	Median [IQR]	*p*	Effect size
Cutting speed [s] (5 mm or smaller vessels)	NGHD	60	2.61 [2.51–2.70]	0.36	2.67 [2.33–2.85]	*p* < 0.01	Cohen's *d* = −1.01
	TBS	60	2.97 [2.87–3.06]	0.36	2.95 [2.72–3.18]		
Cutting speed [s] (5–7 mm vessels)	NGHD	30	2.41 [2.31–2.50]	0.26	2.42 [2.26–2.61]	*p* < 0.01	Cohen's *d* = −2.48
	TBS	30	3.04 [2.94–3.13]	0.25	3.07 [2.81–3.22]		
Burst pressure [mmHg] (5 mm or smaller vessels) The probability was shown in parentheses next to the mean.	NGHD	120	1439.1 (99.4%) [1361.4–1516.9]	430.2	1442.3 [1201.5–1726.9]	N.S.	—
	TBS	120	1314.1 (98.7%) [1242.5–1385.7]	396.1	1335.6 [1015.7–1649.0]		
Burst pressure [mmHg] (5–7 mm vessels) The probability was shown in parentheses next to the mean.	NGHD	60	1303.7 (99.9%) [1228.1–1379.2]	292.5	1277.9 [1058.7–1567.4]	N.S.	—
	TBS	60	1193.8 (95.4%) [1085.4–1302.2]	419.7	1289.2 [998.8–1470.4]		
Burst pressure [mmHg] (5–7 mm vs. PS) The probability was shown in parentheses next to the mean.	NGHD	30	1310.3 (98.0%) [1201.7–1418.9]	290.9	1343.0 [1155.7–1546.3]	N.S.	—
	PS	30	1276.0 (99.9%) [1171.3–1380.7]	280.4	1325.6 [1044.0–1459.6]		
Burst pressure [mmHg] (physical stimulation, control) The probability was shown in parentheses next to the mean.	NGHD	30	1254.2 (98.4%) [1099.2–1403.5]	415.2	1157.0 [975.8–1476.3]	N.S.	—
	TBS	30	1215.8 (99.6%) [1095.0–1336.6]	323.5	1195.8 [971.0–1396.1]		
Burst pressure [mmHg] (physical stimulation, 3 N horizontal) The probability was shown in parentheses next to the mean.	NGHD	30	1114.2 (96.4%) [957.4–1271.1]	420.0	1028.5 [783.3–1389.4]	N.S.	—
	TBS	30	1071.1 (97.0%) [887.0–1147.1]	348.2	1055.5 [832.8–1256.9]		
Burst pressure [mmHg] (physical stimulation, 3 N vertical) The probability was shown in parentheses next to the mean.	NGHD	30	897.5 (90.7%) [745.6–1049.5]	407.0	812.7 [595.1–1106.9]	N.S.	—
	TBS	30	804.1 (89.5%) [671.7–936.4]	354.4	787.4 [507.5–1069.6]		
Burst pressure [mmHg] (physical stimulation, 6 N horizontal) The probability was shown in parentheses next to the mean.	NGHD	30	987.9 (88.7%) [794.2–1181.6]	518.6	896.3 [679.3–1291.2]	N.S.	—
	TBS	30	834.6 (89.6%) [693.7–975.6]	377.5	812.1 [579.0–985.9]		
Burst pressure [mmHg] (physical stimulation, 6 N vertical) The probability was shown in parentheses next to the mean.	NGHD	30	743.8 (85.3%) [607.3–880.3]	365.4	677.2 [525.0–959.3]	N.S.	—
	TBS	30	740.8 (83.2%) [592.8–888.8]	396.4	752.7 [378.3–1007.3]		
Burst pressure [mmHg] (lymphatic vessels) The probability was shown in parentheses next to the mean.	NGHD	30	786.4 (99.3%) [675.1–897.8]	298.2	741.3 [561.1–961.4]	N.S.	—
	TBS	30	651.0 (100.0%) [543.2–758.9]	288.8	623.1 [382.7–902.1]		
Posterior thermal spread [μm] (Porcine small intestine)	NGHD	9	14.70 [0–31.8]	22.2	0 [0–40.9]	*p* < 0.01	Cliff's delta = −1
	TBS	9	123.1 [87.6–158.7]	46.3	120.7 [83.4–152.2]		
Posterior thermal spread [μm] (Porcine liver)	NGHD	9	0	0	0	*p* < 0.01	Cliff's delta = −1
	TBS	9	187.9 [143.1–232.8]	58.4	157.3 [144.1–246.7]		
Posterior thermal spread [μm] (Porcine artery)	NGHD	7	61.0 [12.8–109.2]	52.1	77.0 [0–104.9]	*p* < 0.05	Cohen's *d* = −1.95
	TBS	7	228.6 [127.2–330.0]	109.6	255.3 [125.0–342.3]		
Posterior thermal spread [μm] (during activation)	NGHD	16	5.67 [0–12.84]	13.46	0 [0–0]	*p* < 0.01	Cliff's delta = −0.66
	TBS	16	84.22 [35.34–133.10]	91.74	49.19 [4.42–170.85]		
The number of activations (thermal injury occurs, Full bite)	NGHD	3	3.67 [2.2–5.1]	0.58	4.0 [3.0–4.0]	—	—
	TBS	3	1.0	0	1.0		
The number of activations (thermal injury occurs, Half bite)	NGHD	3	4.33 [2.9–5.8]	0.58	4.0 [4.0–5.0]	—	—
	TBS	3	1.67 [0–4.5]	1.15	1.0 [1.0–3.0]		
The number of activations (thermal injury occurs, Tip bite)	NGHD	3	5.3 [3.9–6.8]	0.58	5.0 [5.0–6.0]	—	—
	TBS	3	1.0	0	1.0		
Lateral thermal spread [mm]	NGHD	40	2.22 [2.06–2.38]	0.50	2.22 [1.77–2.68]	*p* < 0.05	Cohen's *d* = −0.88
	TBS	40	2.79 [2.55–3.03]	0.76	2.63 [2.29–3.13]		
Jaw temperature [°C]	NGHD	30	88.20 [87.11–89.28]	2.91	87.43 [86.35–89.38]	*p* < 0.01	Cliff's delta = −1
	TBS	30	98.75 [97.46–100.04]	3.45	98.78 [95.56–101.52]		
The number of activations (tissue pads depletion)	NGHD	3	325.3 [188.2–462.4]	55.2	341.0 [264.0–371.0]	*p* < 0.05	Cliff's delta = −1
	TBS	3	216.3 [164.2–268.5]	21.0	209.0 [200–240.0]		
Cavitation ratio [%]	NGHD	8	0.91 [0.86–0.96]	0.06	0.94 [0.85–0.96]	*p* < 0.01	Cohen's *d* = −2.81
	TBS	8	1.34 [1.17–1.51]	0.21	1.26 [1.19–1.52]		
Cavitation reach [mm]	NGHD	8	0.49 [0.43–0.56]	0.08	0.45 [0.43–0.57]	*p* < 0.01	Cliff's delta = −1
	TBS	8	1.10 [0.87–1.33]	0.28	1.18 [0.81–1.26]		

Abbreviation: N.S.: not significant.

### 3.1 Cutting Speed

**FIGURE 2:**
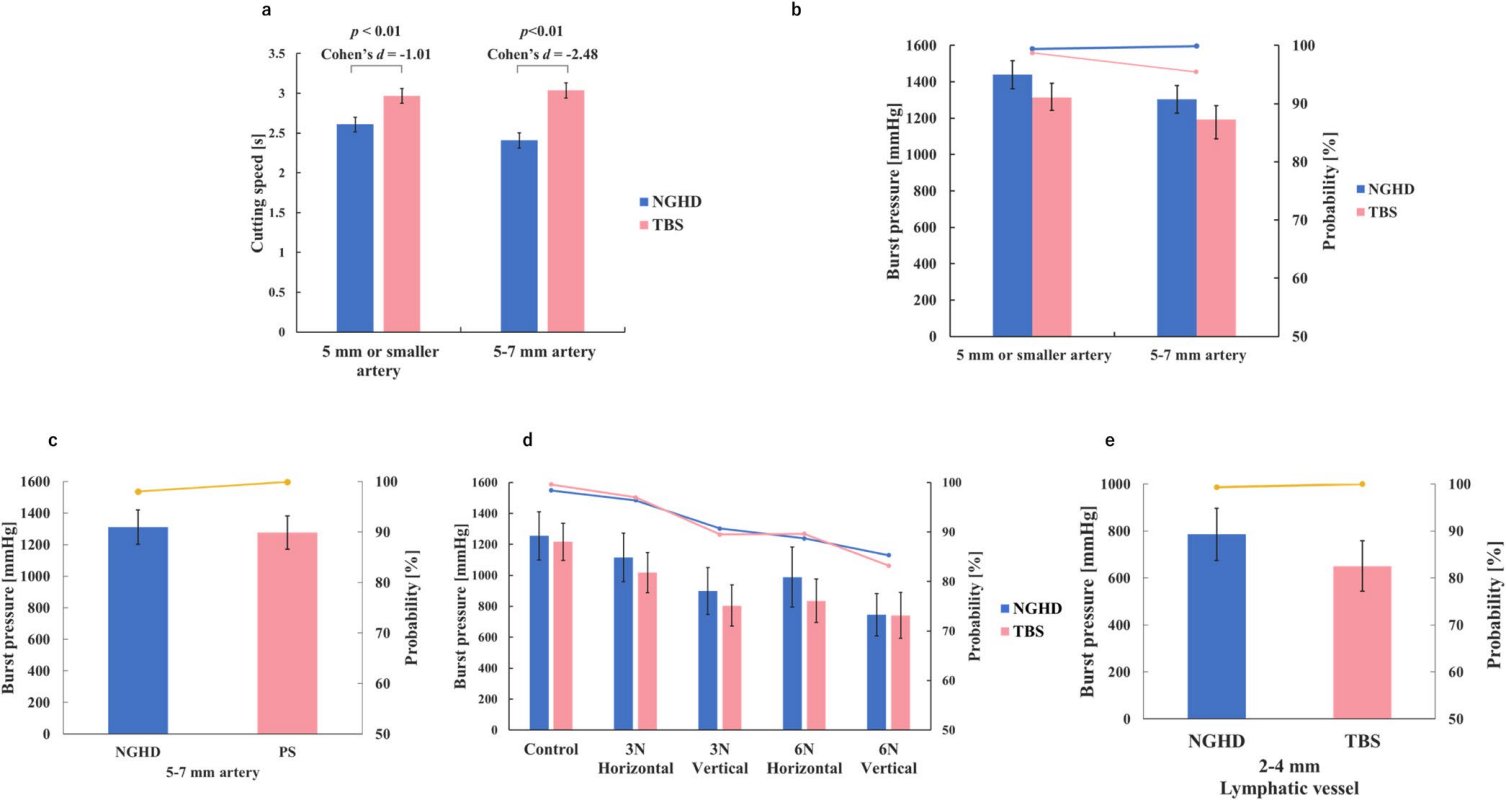
Time required to cut the artery, and burst pressure value when sealing and cutting the artery and lymphatic vessels. (a) Time required for cutting in both vessel types. The bar graphs indicate the mean cutting time, whereas the error bars indicate the 95% confidence interval for the mean. Significant *p* values and effect sizes are also indicated. (b) Burst pressure and probability for each outer diameter. The bar graphs indicate the mean burst pressure, the line graphs indicate the probability, and the error bars indicate the 95% confidence interval for the mean. (c) Burst pressure and probability for 5–7‐mm arteries with NGHD and PS. The bar graphs indicate the mean burst pressure, the line graphs indicate the probability, and the error bars indicate the 95% confidence interval for the mean. (d) Burst pressure and probability for each condition. The bar graphs indicate the mean sealing force, the line graphs indicate the probability, and the error bars indicate the 95% confidence interval for the mean. (e) Burst pressure and probability for the lymphatic vessel. The bar graphs indicate the mean sealing force, the line graphs indicate the probability, and the error bars indicate the 95% confidence interval for the mean. Abbreviations: NGHD, next‐generation hybrid device; TBS, THUNDERBEAT type S.

Cutting speed was evaluated according to vessel diameter. In the vessels with a diameter of 5 mm or smaller, the cutting speed was 2.61 [95% CI: 2.51–2.70] s and 2.97 [2.87–3.06] s with the NGHD and TBS, respectively, indicating a significant difference in dissection speed (*p* < 0.01, Cohen's *d* = −1.01) (*n* = 60 per device). Evaluation of 5–7 mm vessels showed that the cutting speed was 2.41 [2.31–2.50] s and 3.04 [2.94–3.13] s for the NGHD and TBS, respectively, with a significant difference also being observed between devices (*p* < 0.01, Cohen's *d* = −2.48) (*n* = 30 per device) (Figure 2a).

### 3.2 Burst Pressure for the Blood and Lymphatic Vessels

Burst pressure was evaluated according to vessel diameter. Although burst pressure tended to be higher in the NGHD, no significant difference between devices was observed both in 5‐mm (*n* = 120 per device) or smaller vessels and 5–7‐mm vessels (*n* = 60 per device) (Figure 2b). We compared the burst pressure of 5–7‐mm vessels with the NGHD and the advanced bipolar output using NGHD and PS (*n* = 30 per device). Accordingly, no significant difference was observed between the devices (Figure 2c). Given our occasional encounter with delayed bleeding from sealed vessels during surgery, we evaluated the burst pressure while applying physical stimulation to the edge excised by applying S&C. Similarly, no significant differences between the groups were observed (Figure 2d). Evaluation of burst pressures for lymphatic vessels showed that although they tended to be higher in the NGHD, no significant difference was observed between the devices (*n* = 30 per device) (Figure 2e).

### 3.3 Thermal Spread

**FIGURE 3:**
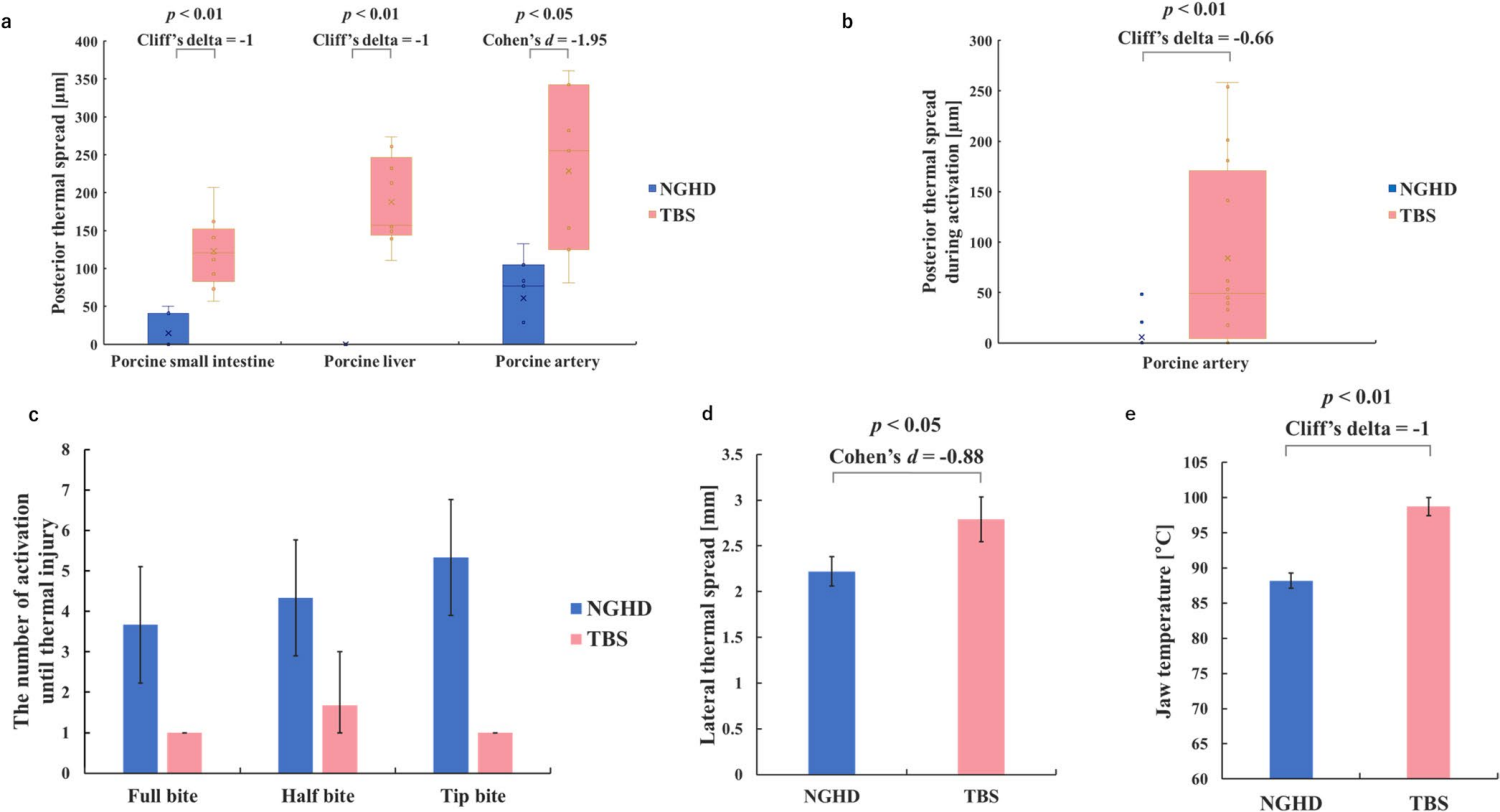
Thermal spread under several conditions. (a) Posterior thermal spreads to each organ. The box plots show posterior thermal spread for each organ. Significant *p* values and effect sizes are also indicated. (b) Posterior thermal spread during the output. The box plots show posterior thermal spread during the output. Significant *p* values and effect sizes are also indicated. (c) Number of activations until thermal injury. The bar graphs indicate the mean number of outputs, whereas the error bars indicate the 95% confidence interval for the mean. (d) Lateral thermal spread. The bar graphs indicate the mean lateral thermal spread, whereas the error bars indicate the 95% confidence interval for the mean. The significant *p* value and the effect size are also indicated. (e) The tip temperature of the device. The bar graphs indicate the mean temperature of the back side of the jaw. The error bars indicate the 95% confidence interval for the mean. The significant *p* value and the effect size are also indicated.

To evaluate the risk of thermal injury during surgery, the thermal spread induced by tissue dissection was examined by simulating specific procedures. Posterior thermal spread through the jaw was assessed based on the adjacent organ. As shown in Figure 3a, a significant reduction in tissue injury for all tested organs was observed with the NGHD (*n* = 9 for small intestine and liver, *n* = 7 for carotid arteries, per device). Next, we evaluated the concurrent thermal damage during activation. The depth of tissue damage toward the artery during activation was significantly deeper with TBS (84.22 [35.34–133.10] μm) than with the NGHD, (5.67 [0–12.84] μm) (*n* = 16 per device) (Figure 3b). Moreover, we evaluated the number of activations until the jaw caused thermal injury to the adjacent organ. Notably, we were able to dissect tissue with the tip bite five times with the NGHD, but only one activation with TBS induced thermal damage (*n* = 3 per device) (Figure 3c). Lateral thermal spread was assessed using porcine mesometrium uteri. As shown in Figure 3d, the length of the lateral thermal spread was significantly shorter with the NGHD (2.22 [2.06–2.38] mm) than with TBS (2.79 [2.55–3.03] mm) (*n* = 40 per device).

As the support data for thermal spread, the temperatures of the device tip were measured. The temperature of the jaw of NGHD was 88.20 [87.11–89.28] °C and lower than TBS 98.75 [97.46–100.04] °C. (*n* = 30 per device) (Figure 3e).

### 3.4 Tissue Pad Durability

**FIGURE 4:**
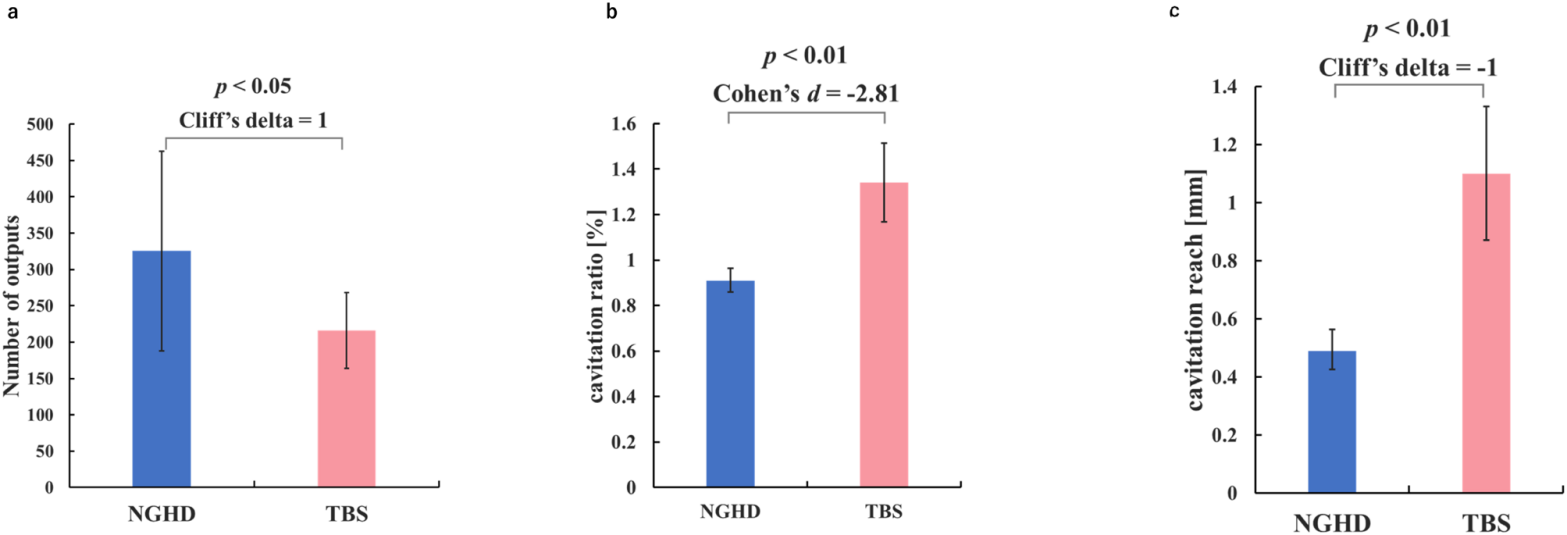
Durability of the tissue pad, cavitation ratio, and reach. (a) Number of outputs until the tissue pad breaks. The bar graphs indicate the mean number of outputs, whereas the error bars indicate the 95% confidence interval for the mean. The significant *p* value and the effect size are also indicated. (b) Cavitation ratio in the imaging range. The bar graphs indicate the mean cavitation ratio, whereas the error bars indicate the 95% confidence interval for the mean. The significant *p* value and the effect size are also indicated. (c) Distance reached by cavitation. The bar graphs indicate the mean cavitation reach, whereas the error bars indicate the 95% confidence interval for the mean. The significant *p* value and the effect size are also indicated. Abbreviations: NGHD, next‐generation hybrid device; TBS, THUNDERBEAT type S.

Tissue pad durability is critical for the safety of endoscopic surgery. Activation without tissue grasping was repeated until the tissue pad was depleted (*n* = 3 per device). The number of activations was significantly higher with the NGHD than with TBS (Figure 4a), indicating a lower risk of equipment failure during surgery in the former.

### 3.5 Mist Production by Cavitation

Mist generated by the cavitation of the ultrasonic probe can interfere with the surgical field. Given the importance of maintaining a clear surgical view in improving the quality of endoscopic surgery, the magnitude of cavitation of the devices was evaluated (*n* = 8 per device). Accordingly, we found that NGHD produced significantly fewer cavitations than did TBS, which implies a reduction in mist production during the procedures (Figure 4b,c).

### 3.6 Perceived Clinical Usefulness

**FIGURE 5:**
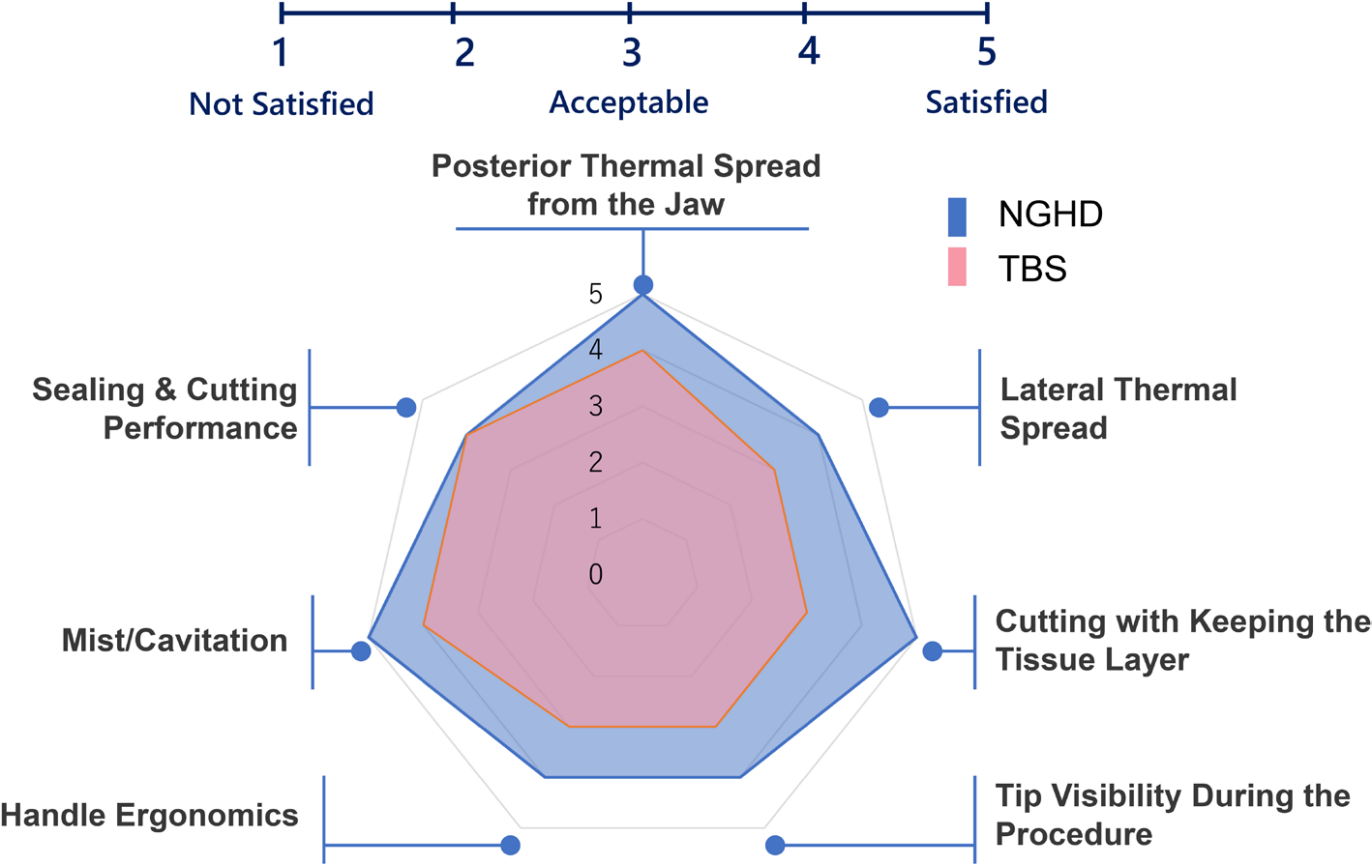
Chart indicating the median scores for each question from seven surgeons. Abbreviations: NGHD, next‐generation hybrid device; TBS, THUNDERBEAT type S.

Seven surgeons simulated the clinical use of the NGHD and TBS in pigs to assess the devices' performance. Performance parameters were rated by all participants on a 5‐point scale (Table 4). As shown in Figure 5, the median score of the NGHD for all questions was equal to or higher than that of the TBS.

**TABLE 4:** Rating for each device by seven surgeons.

Device performance questions	Device	Median (IQR)	Surgeon response score (*n*)				
			<5>	<4>	<3>	<2>	<1>
Rate the degree of posterior thermal spread from the jaw	NGHD	5 (5–5)	7	0	0	0	0
	TBS	4 (3–4)	0	4	3	0	0
Rate the degree of lateral thermal spread	NGHD	4 (4–5)	2	4	1	0	0
	TBS	3 (3–4)	0	3	4	0	0
Rate the capability of cutting while keeping the tissue layer	NGHD	5 (4–5)	5	2	0	0	0
	TBS	3 (3–4)	0	3	4	0	0
Rate the tip visibility during the procedure	NGHD	4 (3–5)	2	3	2	0	0
	TBS	3 (3–4)	0	2	4	0	0
Rate the handle ergonomics	NGHD	4 (4–5)	3	4	0	0	0
	TBS	3 (2–4)	0	2	3	2	0
Rate the amount of mist/cavitation during the activation	NGHD	5 (4–5)	4	3	0	0	0
	TBS	4 (3.75–4)	0	5	1	0	0
Rate the vessel sealing and cutting performance	NGHD	4 (4–5)	3	4	0	0	0
	TBS	4 (4–4)	1	6	0	0	0

*Note:* Responses to each question were scored as follows: 5, satisfied; 4, slightly satisfied; 3, acceptable; 2, slightly not satisfied; 1, not satisfied.

Abbreviations: NGHD, next‐generation hybrid device; TBS, THUNDERBEAT type S.

## 4 Discussion

Energy devices are important for the quick and safe performance of sophisticated procedures during endoscopic surgery. However, such devices have been associated with increased complications depending on their usage [15]. The current study successfully confirmed that the NGHD promotes minimal thermal damage to surrounding tissue, has a durable tissue pad, and has excellent handle ergonomics, allowing it to maintain its advantages in cutting speed and secured sealing over TBS, which has been widely utilized in today's practice given its short cutting time and secure hemostasis. The NGHD can therefore be expected to become a promising surgical instrument for endoscopic surgery, especially in complicated cases involving narrow spaces with prolonged surgical durations.

The efficacy of the NGHD was determined by assessing its cutting speed, burst pressure, and mist generation through the bench‐top study. The cutting capability while maintaining the tissue layer, tip visibility during the procedure, and handle ergonomics were evaluated by surgeons who simulated the use of the NGHD and TBS in pigs. Owing to the narrower top surface of the probe (green arrow in Figure 1b), which allows for effective tissue compression (cross‐sectional view of compressed tissue by the device in Figure 1b), the NGHD yielded a faster cutting speed than did TBS while maintaining the vessel sealing capability. Furthermore, thanks to this tip design, the NGHD achieved a smaller ultrasonic amplitude than did TBS, which reduced cavitation and mist generation during activation, consequently improving the surgical view. The median scores from the surgeons' rating implied that the NGHD was better than TBS in terms of cutting capability while maintaining the tissue layer, tip visibility during the procedure, and handle ergonomics.

With regard to safety, we evaluated the thermal spread to surrounding tissues and the durability of the tissue pad using the bench‐top study. Given that thermal damage has been a major concern of the conventional ultrasonic scissors [16], it was tested under various situations, such as posterior thermal spread from the back of the jaw and lateral thermal spread. Accordingly, our findings showed remarkably minimal posterior thermal spread from the back of the jaw with the NGHD owing to the thermal shield on the jaw. Lateral thermal spread was also reduced with the NGHD given its decreased probe width and bipolar electrode areas (orange arrows shown in Figure 1b) compared to the TBS while maintaining the vessel sealing capability. Moreover, thanks to the decreased ultrasonic amplitude of the NGHD relative to that of TBS, the improvement in the durability of the tissue pad was confirmed. The modified tip structure for combined energy output can help surgeons improve the quality of endoscopic surgery.

Several limitations of the current study need to be acknowledged. First, given that this study performed ex vivo and nonclinical in vivo examinations, the outcomes of the NGHD during surgery on actual patients need to be verified. However, we identified the important factors that need to be considered during surgery, such as hemostatic effect, dissection speed, cavitation, and thermal injury, which were quantitatively evaluated in our ex vivo study and qualitatively evaluated in our nonclinical in vivo study. Second, the ex vivo and nonclinical in vivo experiments with TBS and the NGHD were not blinded, which may have introduced observer bias. However, as described in the Section 2, each experiment was carefully designed to allow quantification. Therefore, the similarities and differences between the devices were evaluated objectively.

In conclusion, the current study confirmed that the NGHD was able to address the concerns associated with TBS while maintaining the benefits of TBS S&C, which combines ultrasonic and advanced bipolar output. These findings suggest that the NGHD has the potential to support safe and efficient endoscopic procedures in clinical settings, pending further clinical evaluation. Regarding future perspectives, we also found room for improvements in the NGHD through our simulation of its clinical use in pigs, such as the thermal control of the probe and improvements in the functionality of the dissector.

## Author Contributions

Satoru Matsuda and Ichiro Uyama were responsible for the concept and design of the study. Jumpei Torikai contributed to the writing of the manuscript and the acquisition and analysis of the data, Sayaka Yasui contributed to the acquisition and analysis of data. Satoru Matsuda wrote the initial draft. All authors participated in developing or reviewing the manuscript and provided final approval for its submission for publication. All authors had full access to all the data in the study and had final responsibility for the decision to submit for publication.

## Funding

This study was conducted in collaboration with Satoru Matsuda, Kazuhisa Ehara, Hiroyasu Kagawa, Shunsuke Tsukamoto, Kohei Nakata, Mamoru Morimoto, Ichiro Uyama and Olympus Medical System Corp. (Tokyo, Japan) based on a contract. The funding for this study was provided by Olympus Medical System Corp.

## Ethics Statement

All procedures performed in vivo were reviewed and approved by the Institutional Animal Care and Use Committee at a Japan Pharmaceutical Information Center‐accredited facility in accordance with the Basic Policy on Animal Experimentation Performed at the Institutions under the Jurisdiction of the Ministry of Health, Labour, and Welfare.

## Conflicts of Interest

Satoru Matsuda, Kazuhisa Ehara, Hiroyasu Kagawa, Shunsuke Tsukamoto, Kohei Nakata, Mamoru Morimoto, and Ichiro Uyama received consulting fees from Olympus Medical Systems Corporation. Jumpei Torikai and Sayaka Yasui are employees of Olympus Medical Systems Corporation.

## Supporting information


**Figure S1:** Experimental setup and method to measure the cutting speed and burst pressures of vessels.
**Figure S2:** Experimental setup and method to measure posterior thermal spread.
**Figure S3:** Experimental setup and method to measure lateral thermal spread.
**Figure S4:** Experimental method to measure tissue pad durability.
**Figure S5:** Experimental setup and method to measure cavitation.

## Data Availability

The data that support the findings of this study are available from the corresponding author upon reasonable request.

## References

[1] H. Katai , J. Mizusawa , H. Katayama , et al., “Survival Outcomes After Laparoscopy‐Assisted Distal Gastrectomy Versus Open Distal Gastrectomy With Nodal Dissection for Clinical Stage IA or IB Gastric Cancer (JCOG0912): A Multicentre, Non‐Inferiority, Phase 3 Randomised Controlled Trial,” Lancet Gastroenterology & Hepatology 5 (2020): 142–151. 31757656 10.1016/S2468-1253(19)30332-2

[2] S. Kitano , M. Inomata , J. Mizusawa , et al., “Survival Outcomes Following Laparoscopic Versus Open D3 Dissection for Stage II or III Colon Cancer (JCOG0404): A Phase 3, Randomised Controlled Trial,” Lancet Gastroenterology & Hepatology 2 (2017): 261–268. 28404155 10.1016/S2468-1253(16)30207-2

[3] K. Kataoka , H. Takeuchi , J. Mizusawa , et al., “A Randomized Phase III Trial of Thoracoscopic Versus Open Esophagectomy for Thoracic Esophageal Cancer: Japan Clinical Oncology Group Study JCOG1409,” Japanese Journal of Clinical Oncology 46 (2016): 174–177. 26732383 10.1093/jjco/hyv178

[4] H. Takeuchi , M. Ando , Y. Tsubosa , et al., “A Randomized Controlled Phase III Trial Comparing Thoracoscopic Esophagectomy and Open Esophagectomy for Thoracic Esophageal Cancer: JCOG1409 (MONET Trial),” Journal of Clinical Oncology 42 (2024): 249.

[5] S. Ito , A. Takahashi , H. Ueno , et al., “Annual Report on National Clinical Database 2021 for Gastroenterological Surgery in Japan,” Annals of Gastroenterological Surgery 9 (2025): 32–59. 39759995 10.1002/ags3.12868 PMC11693552

[6] J. C. Taylor , D. Burke , L. H. Iversen , et al., “Minimally Invasive Surgery for Colorectal Cancer: Benchmarking Uptake for a Regional Improvement Programme,” Clinical Colorectal Cancer 23 (2024): 382–391.e1. 39004595 10.1016/j.clcc.2024.05.013

[7] S. Matsuda , P. van der Sluis , H. Kumamaru , et al., “Oesophagectomy in the East Versus the West: Comparison of Two National Audit Databases,” British Journal of Surgery 112 (2025): znaf035, 10.1093/bjs/znaf035. 40265485 PMC12015469

[8] T. Gao , B. E. Lau , T. Yamaguchi , et al., “Experimental Analyses of the Cavitation Generated by Ultrasonically Activated Surgical Devices,” Surgery Today 47 (2017): 122–129. 27154459 10.1007/s00595-016-1345-1

[9] K. Shibao , F. Joden , and Y. Adachi , “Repeated Partial Tissue Bite With Inadequate Cooling Time for an Energy Device May Cause Thermal Injury,” Surgical Endoscopy 35, no. 6 (2021): 3189–3198. 33523265 10.1007/s00464-021-08322-3

[10] M. Tahir and W. Gilkison , “Ureteric Injury due to the Use of LigaSure,” Case Reports in Urology 2013 (2013): 989524. 24171134 10.1155/2013/989524 PMC3792546

[11] J. W. Milsom , K. Trencheva , T. Sonoda , G. Nandakumar , P. J. Shukla , and S. Lee , “A Prospective Trial Evaluating the Clinical Performance of a Novel Surgical Energy Device in Laparoscopic Colon Surgery,” Surgical Endoscopy 29 (2015): 1161–1166. 25159634 10.1007/s00464-014-3783-4

[12] D. C. Steinemann , S. H. Lamm , and A. Zerz , “Efficacy and Safety of Combined Ultrasonic and Bipolar Energy Source in Laparoscopic Surgery,” Journal of Gastrointestinal Surgery 20, no. 10 (2016): 1760–1768. 27456017 10.1007/s11605-016-3217-9

[13] R. Chambers , D. Sarno , and S. Roweton , “New Bipolar Electrosurgical Vessel Sealing Device Provides Improved Performance and Procedural Efficiency,” Medical Devices: Evidence and Research 18 (2025): 75–86. 39882537 10.2147/MDER.S498873 PMC11776930

[14] J. In and D. K. Lee , “Alternatives to the P Value: Connotations of Significance,” Korean Journal of Anesthesiology 77, no. 3 (2024): 316–325. 38835136 10.4097/kja.23630 PMC11150123

[15] T. Homma , “Advances and Safe Use of Energy Devices in Lung Cancer Surgery,” General Thoracic and Cardiovascular Surgery 70 (2022): 207–218. 35107778 10.1007/s11748-022-01775-w PMC8881425

[16] S. Takahashi , N. Gotohda , Y. Kato , and M. Konishi , “Measure of Pancreas Transection and Postoperative Pancreatic Fistula,” Journal of Surgical Research 202 (2016): 276–283. 27229101 10.1016/j.jss.2016.01.008

