# Supplementary material for: Preclinical Evaluation of a New Surgical Hybrid Energy Device Compared to the Conventional Energy Device
Source: Asian J Endosc Surg. 2025 Nov 27;18(1):e70201. doi: 10.1111/ases.70201 (PMC12661100; doi:10.1111/ases.70201)
Supplement: Supplementary file 1 — Figure S1: Experimental setup and method to measure the cutting speed and burst pressures of vessels. Figure S2: Experimental setup and method to measure posterior thermal spread. Figure S3: Experimental setup and method to measure lateral thermal spread. Figure S4: Experimental method to measure tissue pad durability. Figure S5: Experimental setup and method to measure cavitation. [file ASES-18-e70201-s001.pptx]

## Slide 1
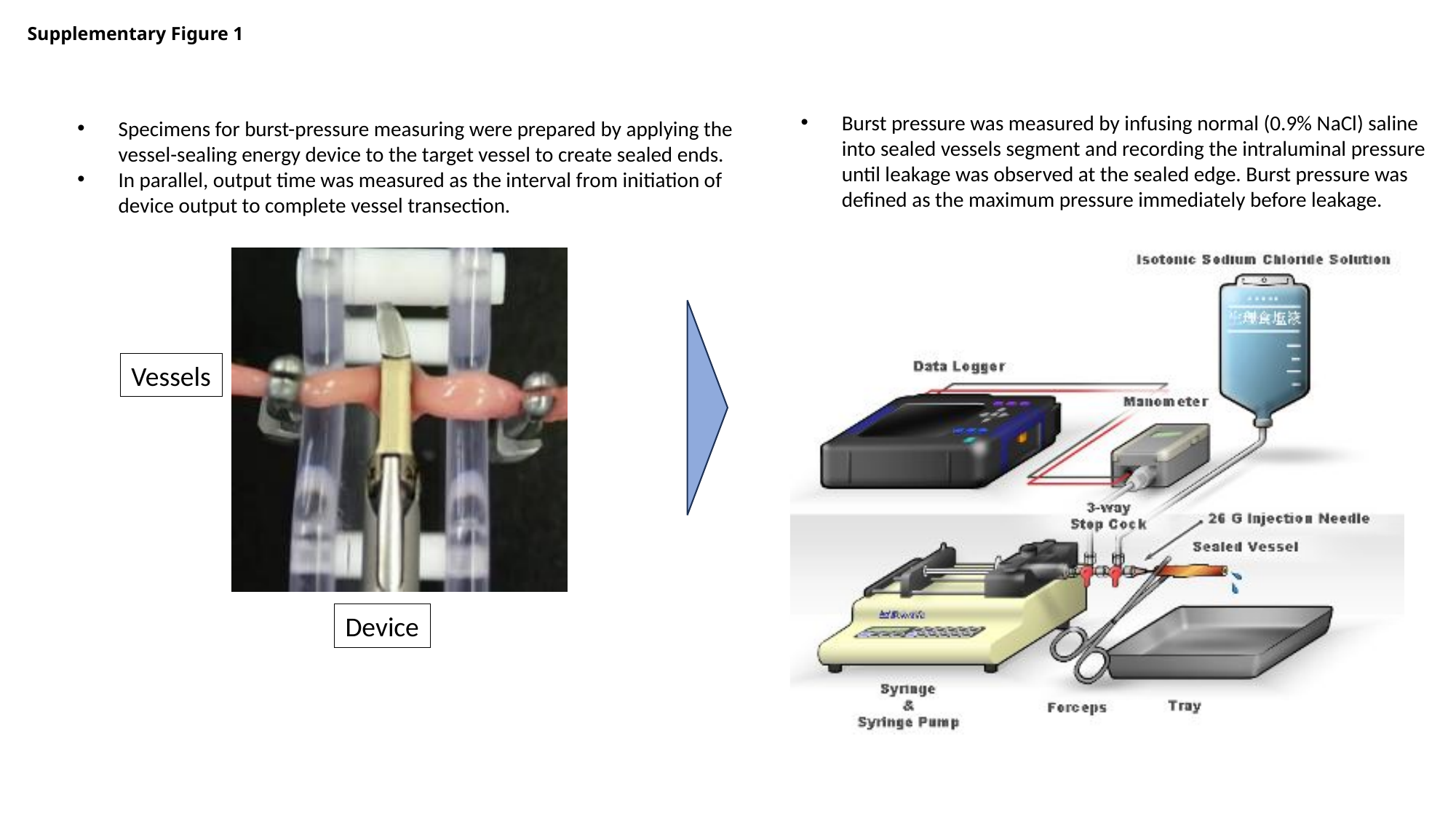

Supplementary Figure 1
Burst pressure was measured by infusing normal (0.9% NaCl) saline into sealed vessels segment and recording the intraluminal pressure until leakage was observed at the sealed edge. Burst pressure was defined as the maximum pressure immediately before leakage.
Specimens for burst-pressure measuring were prepared by applying the vessel-sealing energy device to the target vessel to create sealed ends.
In parallel, output time was measured as the interval from initiation of device output to complete vessel transection.
Vessels
Device

## Slide 2
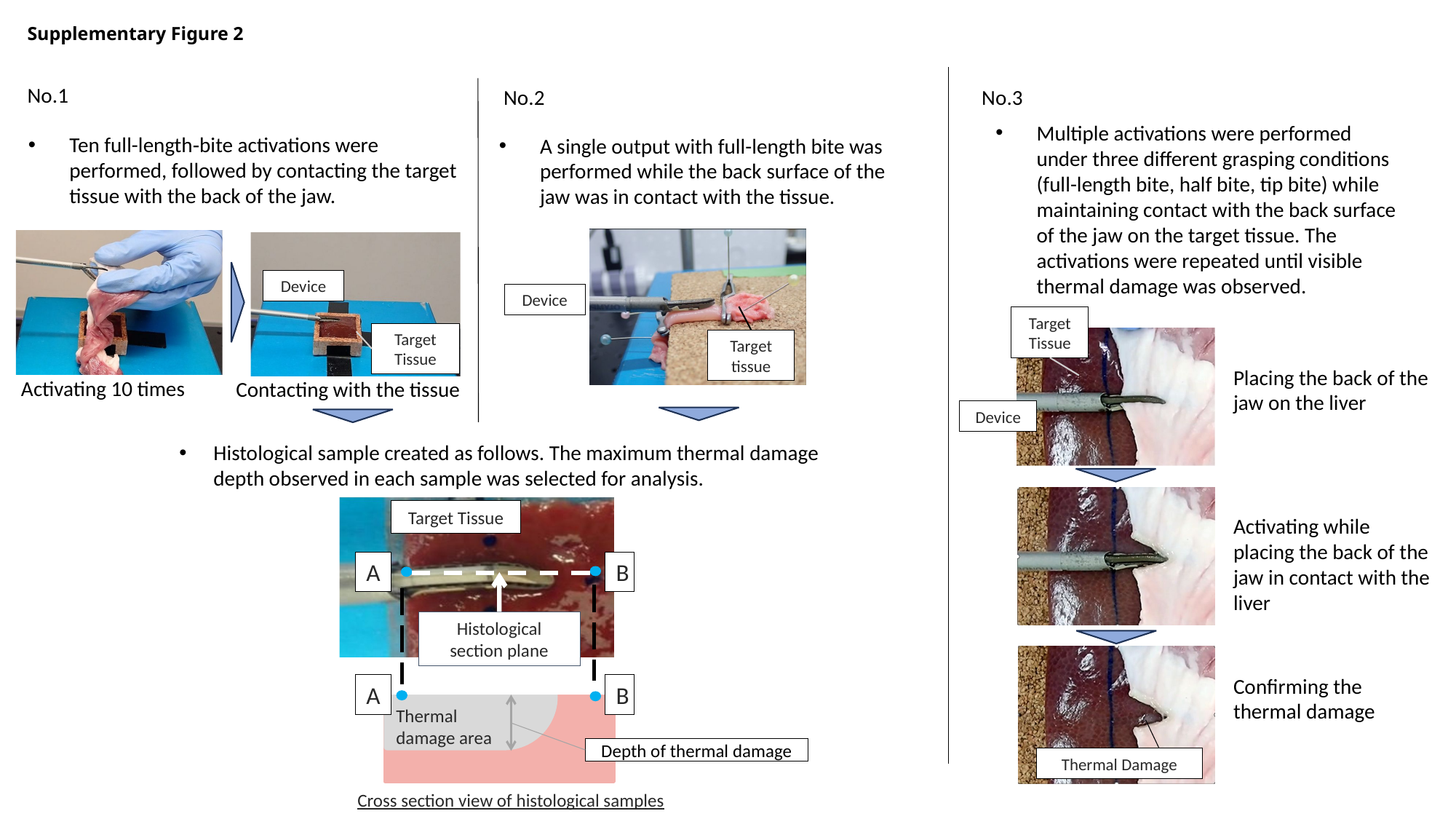

Supplementary Figure 2
No.1
No.2
No.3
Multiple activations were performed under three different grasping conditions (full-length bite, half bite, tip bite) while maintaining contact with the back surface of the jaw on the target tissue. The activations were repeated until visible thermal damage was observed.
Ten full-length‑bite activations were performed, followed by contacting the target tissue with the back of the jaw.
A single output with full-length bite was performed while the back surface of the jaw was in contact with the tissue.
Device
Device
Target Tissue
Target
Tissue
Target tissue
Placing the back of the jaw on the liver
Activating 10 times
Contacting with the tissue
Device
Histological sample created as follows. The maximum thermal damage depth observed in each sample was selected for analysis.
Target Tissue
A
B
Histological section plane
A
B
Thermal damage area
Depth of thermal damage
Cross section view of histological samples
Activating while placing the back of the jaw in contact with the liver
Confirming the thermal damage
Thermal Damage

## Slide 3
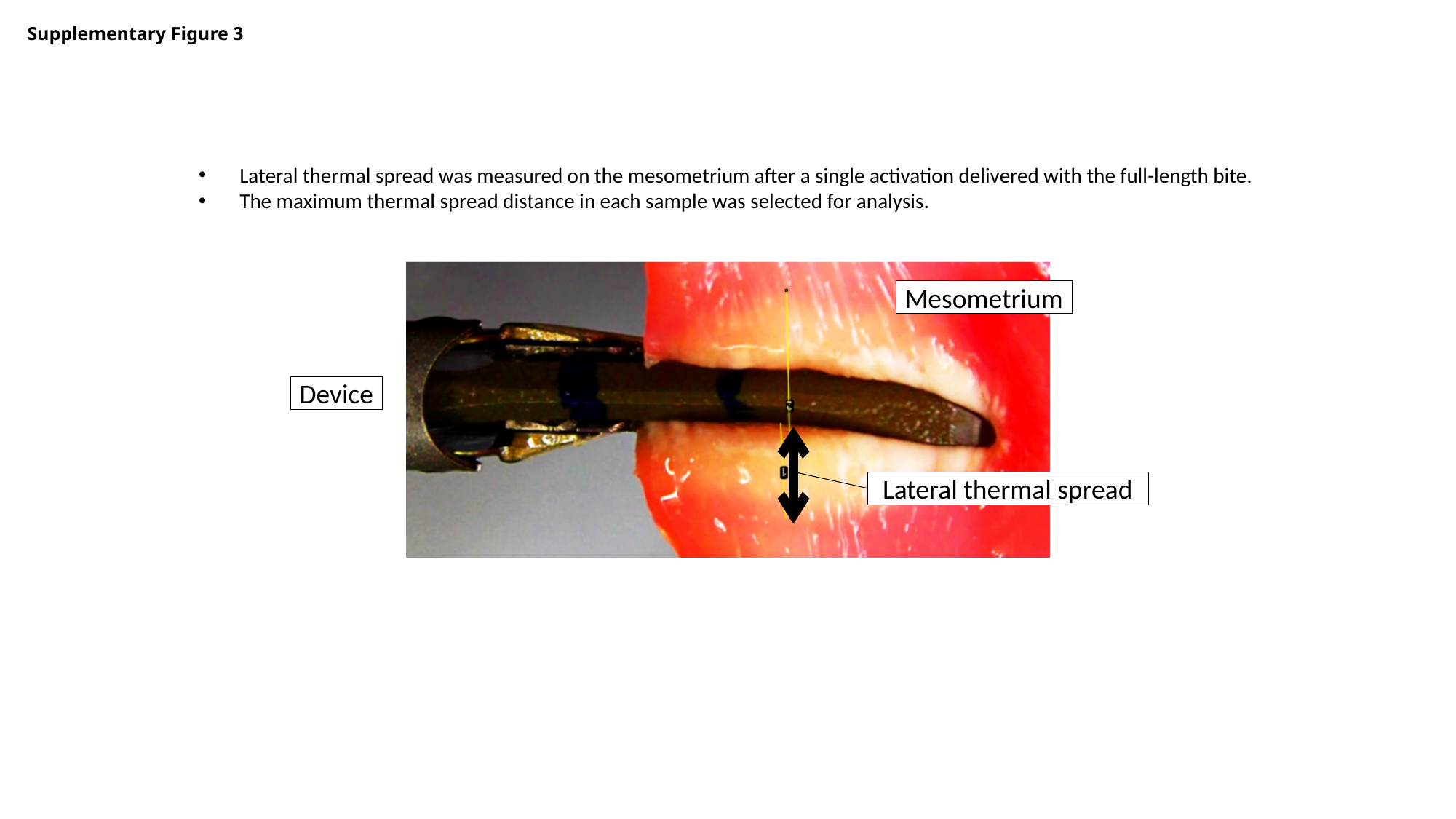

Supplementary Figure 3
Lateral thermal spread was measured on the mesometrium after a single activation delivered with the full-length bite.
The maximum thermal spread distance in each sample was selected for analysis.
Mesometrium
Device
Lateral thermal spread

## Slide 4
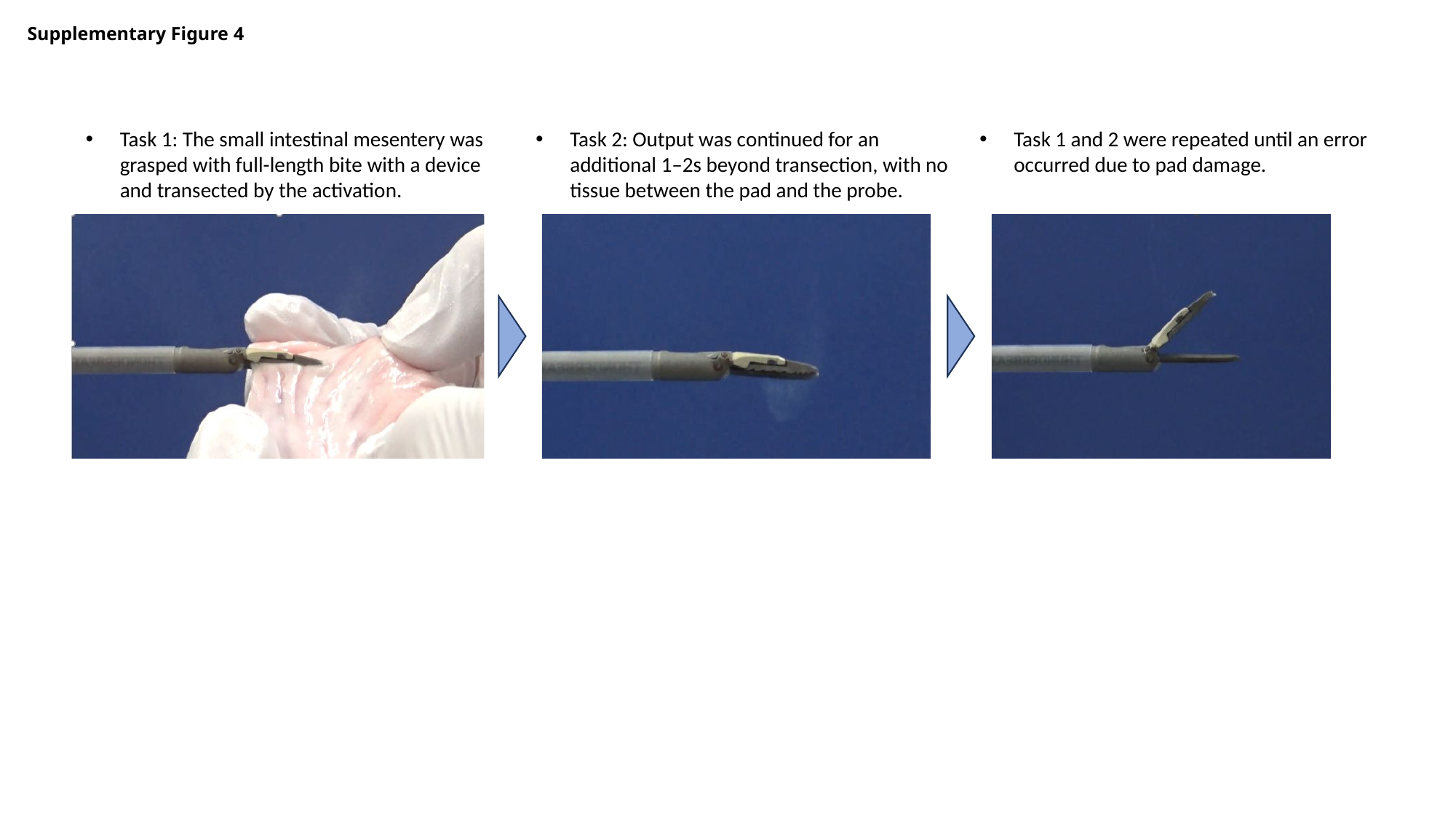

Supplementary Figure 4
Task 1 and 2 were repeated until an error occurred due to pad damage.
Task 1: The small intestinal mesentery was grasped with full-length bite with a device and transected by the activation.
Task 2: Output was continued for an additional 1–2s beyond transection, with no tissue between the pad and the probe.

## Slide 5
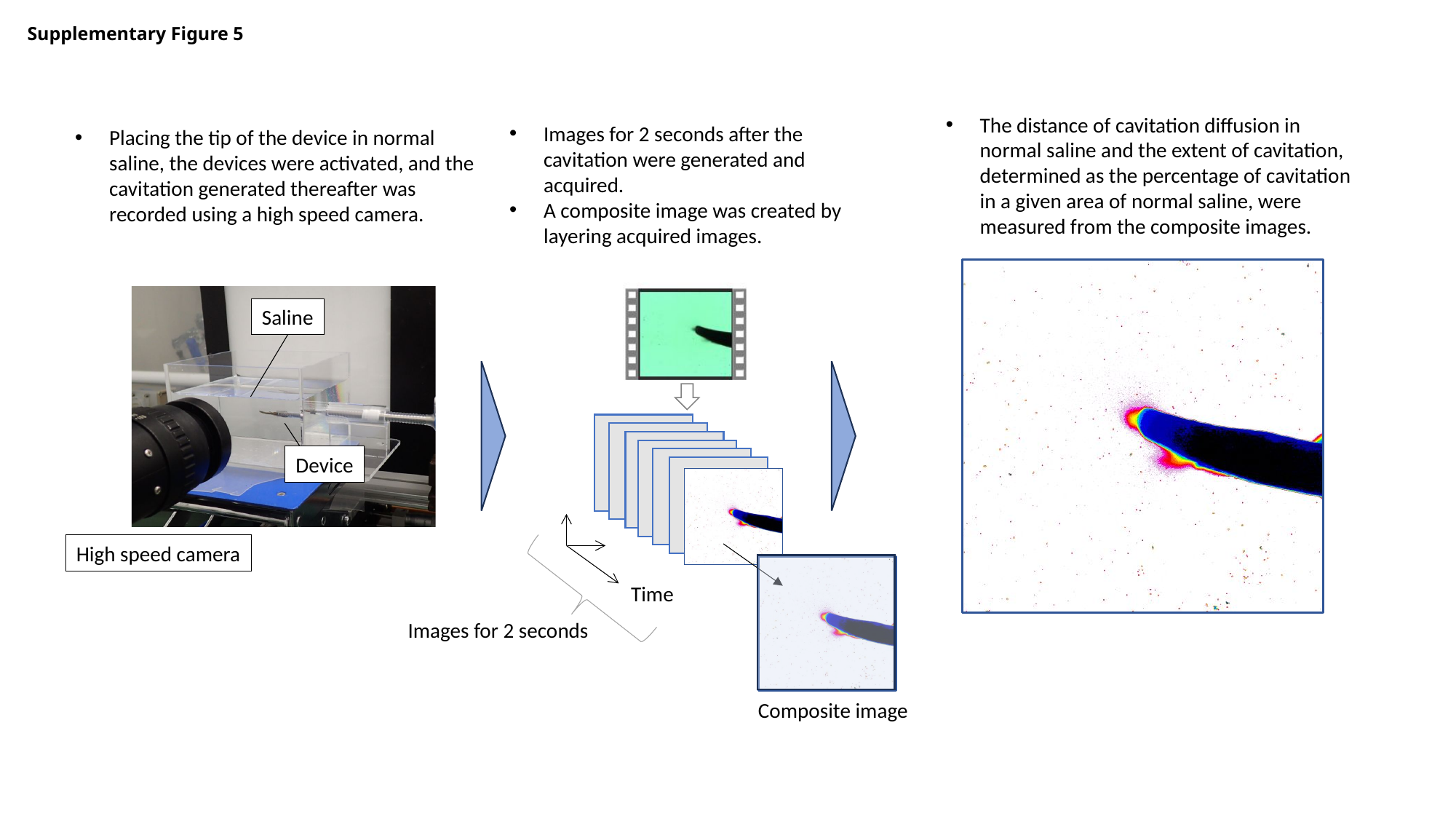

Supplementary Figure 5
The distance of cavitation diffusion in normal saline and the extent of cavitation, determined as the percentage of cavitation in a given area of normal saline, were measured from the composite images.
Images for 2 seconds after the cavitation were generated and acquired.
A composite image was created by layering acquired images.
Placing the tip of the device in normal saline, the devices were activated, and the cavitation generated thereafter was recorded using a high speed camera.
Saline
Device
Time
High speed camera
Images for 2 seconds
Composite image
